# Asymmetric U-shaped association between hypertension and wearable-derived sleep duration

**DOI:** 10.3389/fpubh.2025.1724251

**Published:** 2026-01-12

**Authors:** Haiqi Song, Yunyi Huang, Xiang Fang, Fanyu Lin, Bin Luo, Miaomiao Wu, Xiaoyang Liao, Can Shen, Rong Yang

**Affiliations:** 1 Department of General Internal Medicine, West China Second University Hospital, Sichuan University, Chengdu, China; 2 Key Laboratory of Birth Defects and Related Diseases of Women and Children (Sichuan University), Ministry of Education, Chengdu, China; 3 Wuhan Union Medical College Chongqing Hospital, Tongji Medical College, Huazhong University of Science and Technology, Chengdu, China; 4 General Practice Ward/International Medical Center Ward, Teaching and Research Section of General Practice, General Practice Research Institute, and General Practice Medical Center, West China Hospital, Sichuan University, Chengdu, China

**Keywords:** sleep duration, wearable, hypertension, blood pressure, general public

## Abstract

**Objective:**

To investigate associations between hypertension and wearable-derived sleep duration, including Sleep Period Time (SPT) and Total Sleep Time (TST), in community-dwelling adults.

**Methods:**

In this cross-sectional study, nocturnal SPT and TST were objectively quantified from single-lead electrocardiograms and analyzed using multimodal regression approaches including covariate-adjusted binary logistic regression, restricted cubic splines, and segmented linear regression.

**Results:**

Our analysis of 1,459 valid sleep records from 759 participants showed asymmetric U-shaped associations between objective sleep duration and both hypertension prevalence and blood pressure levels. After adjusting for confounding factors, participants with SPT < 6 h had a 2.05-fold higher prevalence of hypertension (95% CI: 1.18–3.54), while those with TST ≥ 8 h had a 1.82-fold increased odds (95% CI: 1.03–3.22). For SPT, blood pressure nadirs occurred at 7.6–7.7 h for both systolic blood pressure (SBP) and diastolic blood pressure (DBP). For TST, the optimal duration was 7.0–7.2 h in different models. Below optimal SPT thresholds, each 1-h increase was associated with a reduction of 2.94 mmHg in SBP (95% CI: −4.82 to −1.06) and 1.21 mmHg (95% CI: −2.05 to −0.36) in DBP. In partially adjusted models, each 1-h increase above the optimal SPT was associated with an increase in SBP of 2.27 mmHg (95% CI: 0.15 to 4.39). This effect was no longer significant in the fully adjusted analysis. For TST, similar dose–response patterns were significant only for DBP, in partially adjusted models.

**Conclusion:**

Wearable-derived objective sleep duration demonstrates an asymmetric U-shaped association with both hypertension prevalence and blood pressure parameters. Blood pressure nadirs occurred at 7.6–7.7 h for SPT and 7.0–7.2 h for TST.

## Introduction

Hypertension remains a leading contributor to global disability and mortality, and the predominant risk factor for cardiovascular disease (1, 2). Accelerated urbanization and intensified societal rhythms are making inappropriate sleep duration an emerging modifiable risk factor for hypertension (3, 4). Large-scale epidemiological studies reveal significantly higher hypertension risks at both extremes of sleep duration, with the lowest and highest quartiles demonstrating 23 and 32% increased risks, respectively (5). Consequently, precise sleep monitoring plays a key role in the prevention, management, and prognostic improvement of hypertension (6).

Current sleep monitoring methods present significant limitations (7, 8). In research on sleep and hypertension, most studies still rely on subjective sleep questionnaires, despite evidence that they do not accurately reflect objective sleep measures (9–12). Among objective sleep monitoring methods, polysomnography (PSG) is the most widely used tool. However, especially in the general population, it has limitations including discomfort, sleep disruption, and high cost, which limit its accuracy and usefulness (12, 13). Actigraphy, the most widely used portable sleep monitoring tool, has limitations including reduced classification accuracy and inability to classify sleep stages (12–15). Wearable devices that monitor physiological signals are emerging as a promising tool for objective sleep assessment. Among these physiological signals, heart rate variability (HRV) is an easily accessible biomarker derived from consumer-grade wearables. Novel sleep monitoring technologies are advancing rapidly, with HRV-based sleep staging achieving classification accuracy ranging from 72.8 to 89.8% (16–20). Compared to PSG and actigraphy, HRV-derived sleep analysis offers lower cost, richer multidimensional data, multi-night assessment capability, and higher accuracy in general public (19, 21).

Collectively, these advantages make HRV-Derived sleep parameters a promising tool for sleep and hypertension research, with strong potential to advance this field. Although many studies have established the associations between hypertension and sleep metrics from conventional monitoring tools, it remains unclear whether HRV-derived sleep duration yields comparable or divergent results (22–26). This study aims to examine the associations between HRV-derived sleep duration and both hypertension status and blood pressure profiles.

## Materials and methods

### Study design and setting

This cross-sectional study analyzed data from the 12-year follow-up wave of the Chengdu (China) cohort of the Prospective Urban Rural Epidemiology (PURE) study. The design of the PURE study has been previously published (27, 28). The Chengdu cohort employed cluster sampling to recruit one urban and one rural community based on socioeconomic status. From 2008 to 2010, the study enrolled 2,032 adults aged 35–70 years (1,015 urban residents and 1,017 rural residents). The 12-year follow-up was completed on August 30, 2021, with 1,560 participants retained in the study. All participants provided written informed consent. During the 12-year follow-up of the PURE Chengdu (China) study, we provided single-lead ECG devices to participants who provided additional informed consent. We subsequently combined this single-lead ECG-derived data with the PURE Chengdu (China) dataset. The study protocol was granted ethical approval by the Ethics Committee on Biomedical Research of West China Hospital, Sichuan University (Approval No. 2021 Review (702)). Reporting adhered to the STROBE guidelines for cross-sectional studies (29).

### Participants

In addition to the original PURE study criteria, participants were required to satisfy the following criteria: (1) complete the 12-year follow-up survey of the PURE study; (2) provide supplemental informed consent for this sub-study; and (3) wear the study-provided single-lead ECG device for at least one night. Participants were excluded due to insufficient data completeness (nocturnal recording integrity < 70%) or incomplete covariate documentation.

Hypertension was defined as satisfying any of the following criteria: (1) self-reported history of hypertension in follow-up questionnaires; (2) self-reported current use of antihypertensive medication; and (3) average SBP ≥ 140 mmHg and/or DBP ≥ 90 mmHg from three consecutive readings during standardized physical examinations.

### Data measurement

Data on demographic and clinical characteristics were collected through structured questionnaires and physical examinations as part of the PURE study. The questionnaires collected data on demographic factors (name, sex, date of birth, residence area, educational level, and occupation) and clinical information (medical history, family history, physical activity, smoking status, and alcohol consumption). Physical assessments included blood pressure measurements and anthropometric measurements (height, weight, waist circumference, and hip circumference). All measurements were performed by certified investigators using standardized protocols and the Omron HEM-757 electronic blood pressure monitor in accordance with China’s Guidelines for the Management of Hypertension (30).

Objective sleep duration was derived through analysis of HRV acquired via a single-lead electrocardiogram (ECG). The placement, retrieval of and data collection from the single-lead ECG devices were performed by trained investigators. Following completion of the questionnaires and physical examinations, investigators attached a flexible electrode patch to each participant’s mid-sternum. The device was then paired with a smartphone via Bluetooth, and ECG recording was initiated through the study’s dedicated software. Participants were instructed to maintain their usual sleep patterns during at least one night of nocturnal monitoring before returning the device. This study utilized HRV data from the single-lead ECG devices, wherein the signals were converted into sleep duration parameters using an HRV-based sleep staging model developed by Synwing Tech. This model was trained and internally validated using PSG and ECG data from the MIT-BIH database (31, 32). External validation was conducted using comprehensive portable PSG data from the Sleep Medicine Center of West China Hospital, Sichuan University. Internal validation yielded a sensitivity of 88.4% and a specificity of 82.0%. External validation yielded the best performance with a sensitivity of 81.5% and a specificity of 77.8%.

Sleep parameters were operationally defined as follows: SPT is defined as the total duration from sleep onset to final awakening, encompassing all sleep stages and intermittent wakefulness; TST is defined as the cumulative duration of actual sleep states within SPT, calculated as SPT minus wakefulness after sleep onset, thereby reflecting the pure sleep duration without interruptions. SPT and TST were each stratified into consecutive 1-h intervals, with the 7-8 h SPT group and 6-7 h TST groups designated as references groups. The Apnea-Hypopnea Index (AHI) is defined as the number of apnea-hypopnea events per hour of sleep. The Sleep Midpoint (SMP) is defined as the median clock time between the time of sleep onset and final awakening.

All field investigators received standardized training on the administration of structured questionnaires, conducting physical measurements, and operation of wearable ECG device prior to study initiation. Following data collection, dual data entry was performed by trained investigators via EpiData 3.1. Specifically, two independent investigators entered all questionnaire data, which were then cross-checked. Any discrepancies identified were resolved by a third investigator. The complete dataset was securely maintained at the National Institute of Cardiovascular Diseases, China.

### Statistical analysis

Preliminary data screening for outlier was performed using the interquartile range method. Multivariable-adjusted binary logistic regression analysis was performed to assess the associations between sleep duration and the prevalence of hypertension. Nonlinear relationships between sleep duration and blood pressure parameters were assessed using restricted cubic splines. Segmented linear regression models were employed to quantify the relationships between sleep duration and blood pressure parameters. We constructed three progressively adjusted models: Model 1 adjusted for demographic factors (sex, age, residential area, and education level); Model 2 additionally adjusted for lifestyle and metabolic factors (BMI, smoking status, alcohol consumption, diabetes); and Model 3 further adjusted for sleep parameters (AHI, and sleep midpoint). Statistical significance was defined as two-sided *p* < 0.05. All statistical analyses were conducted using R software (Version 5.4).

## Results

### Participants

From 1,560 participants in the 12-year follow-up wave of the PURE Chengdu (China) Cohort, 759 were enrolled in the present cross-sectional study (Supplementary Figure 1). No statistically significant differences were observed in demographic characteristics, clinical features, or sleep parameters between the included and excluded groups (Supplementary Table 1). Of the included participants, 308 were diagnosed with hypertension, and 451 without. The groups were comparable in terms of demographic characteristics. No statistically significant differences were observed in age, sex distribution, smoking prevalence, or alcohol consumption. Compared with the non-hypertensive control group, participants with hypertension had significantly higher adiposity-related measures, including body mass index (BMI), waist circumference, waist-to-hip ratio, and waist-to-height ratio (*p* < 0.001) (Table 1).

**Table 1:** Comparative analysis of clinical and sleep parameters stratified by hypertension status.

Variables	Total	Without hypertension	With hypertension	*p* value
Participates, *n*	759	308	451	
Age, years	65.83 ± 10.40	65.30 ± 10.88	66.20 ± 10.07	0.247
Gender				
Men, *n* (%)	276 (36.4)	107 (34.7)	169 (37.4)	0.091
Women, *n* (%)	483 (63.6)	201 (65.3)	282 (62.5)	
BMI, kg/m^2^	23.96 ± 2.86	23.96 ± 2.86	25.21 ± 3.47	<0.001
WC, cm	83.63 ± 9.08	81.60 ± 8.22	85.21 ± 9.41	<0.001
WHpR	0.90 ± 0.08	0.89 ± 0.77	0.91 ± 0.76	<0.001
WHtR	0.54 ± 0.06	0.53 ± 0.57	0.55 ± 0.63	<0.001
Blood pressure				
Systolic	138.42 ± 18.99	123.40 ± 10.14	148.67 ± 16.66	<0.001
Diastilic	78.13 ± 10.19	74.30 ± 7.51	80.75 ± 10.93	<0.001
Monitoring times, nights	1,459			
One night, *n* (%)	166 (21.9)			
Two nights, *n* (%)	525 (69.2)			
≥3 nights, *n* (%)	68 (9.0)			
SPT, hours	7.75 ± 1.60	7.85 ± 1.46	7.68 ± 1.69	0.146
<6, *n* (%)	102	39 (38.2)	63 (61.8)	<0.001
6–7, *n* (%)	118	61 (51.7)	57 (48.3)	
7–8, *n* (%)	159	88 (55.3)	71 (44.7)	
8–9, *n* (%)	223	120 (53.8)	103 (46.2)	
>9, *n* (%)	157	73 (46.5)	84 (53.5)	
TST, hours	5.92 ± 1.65	5.85 ± 1.60	5.97 ± 1.69	0.321
<4, *n* (%)	108	52 (48.1)	56 (51.9)	<0.001
4–5, *n* (%)	103	49 (47.6)	54 (52.4)	
5–6, *n* (%)	161	84 (52.2)	77 (47.8)	
6–7, *n* (%)	165	90 (54.5)	75 (45.5)	
7–8, *n* (%)	153	79 (51.6)	74 (48.4)	
≥8, *n* (%)	69	27 (39.1)	42 (60.9)	
AHI, times/h	11.00 (5.75, 18.63)	9.12 (5.40, 17.51)	12.49 (6.03, 20.07)	0.039
<5, *n* (%)	163 (21.5)	69 (22.4)	94 (20.8)	<0.001
5–15, *n* (%)	326 (43.0)	143 (46.4)	183 (40.6)	
15–30, *n* (%)	192 (25.3)	73 (23.7)	119 (26.4)	
≥30, *n* (%)	78 (10.3)	23 (7.5)	55 (12.2)	
SMP, hours	2.78 ± 0.83	2.67 ± 0.81	2.86 ± 0.84	0.003

Data are presented as mean ± standard deviation or median (interquartile range) for continuous variables, and number (percentage) for categorical variables. BMI, body mass index; WC, waist circumference; WHpR, waist-to-hip ratio; WHtR, waist-to-height ratio; SPT, sleep period time; TST, total sleep time; AHI, apnea-hypopnea index; SMP, sleep midpoint. *p*-values derived from independent t-tests, Mann–Whitney U tests, or *χ*^2^ tests as appropriate.

### Sleep monitoring characteristics

Among all participants, 21.9% provided data for one night of sleep monitoring, 69.2% for two nights, and 9.0% for three or more nights, resulting in a total of 1,459 valid sleep recordings. Compared with the non-hypertensive group, the hypertension group had a higher AHI (12.49 vs. 9.12 events/h; *p* < 0.001), and later sleep midpoint (02:52 vs. 02:40; *p* = 0.003). No statistically significant differences were observed both in SPT (7.85 ± 1.46 vs. 7.68 ± 1.69 h; *p* = 0.146) and TST (5.97 ± 1.69 vs. 5.85 ± 1.60 h; *p* = 0.321) between groups (Table 1).

### Association between sleep duration and hypertension prevalence

**Figure 1:**
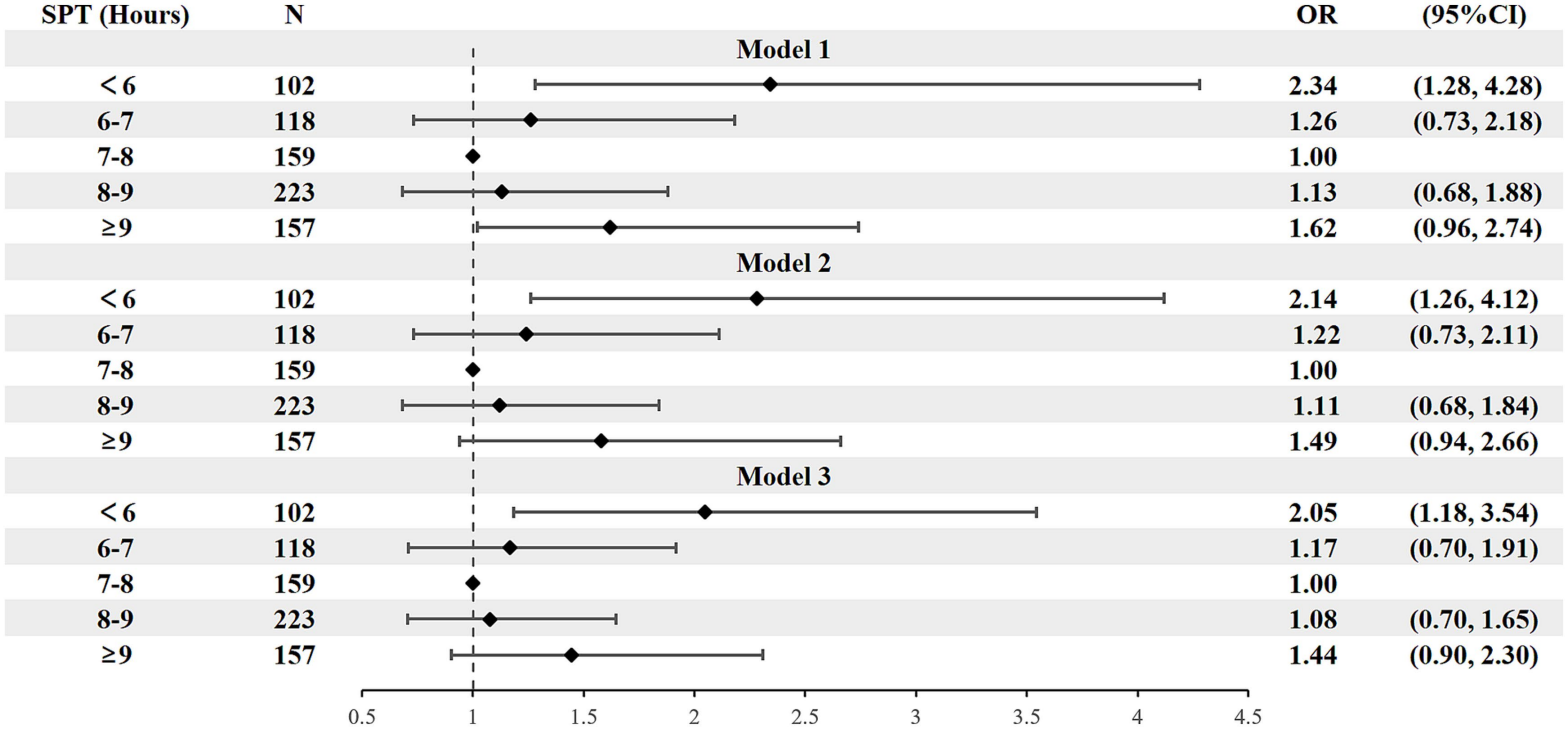
Forest plot of stepwise logistic regression analysis of SPT hypertension prevalence. SPT, sleep period time. Model 1 adjusted for sex, age, residential area, and education level; Model 2 further adjusted for BMI, smoking status, alcohol consumption, and diabetes history; Model 3 additionally adjusted for AHI and sleep midpoint.

**Figure 2:**
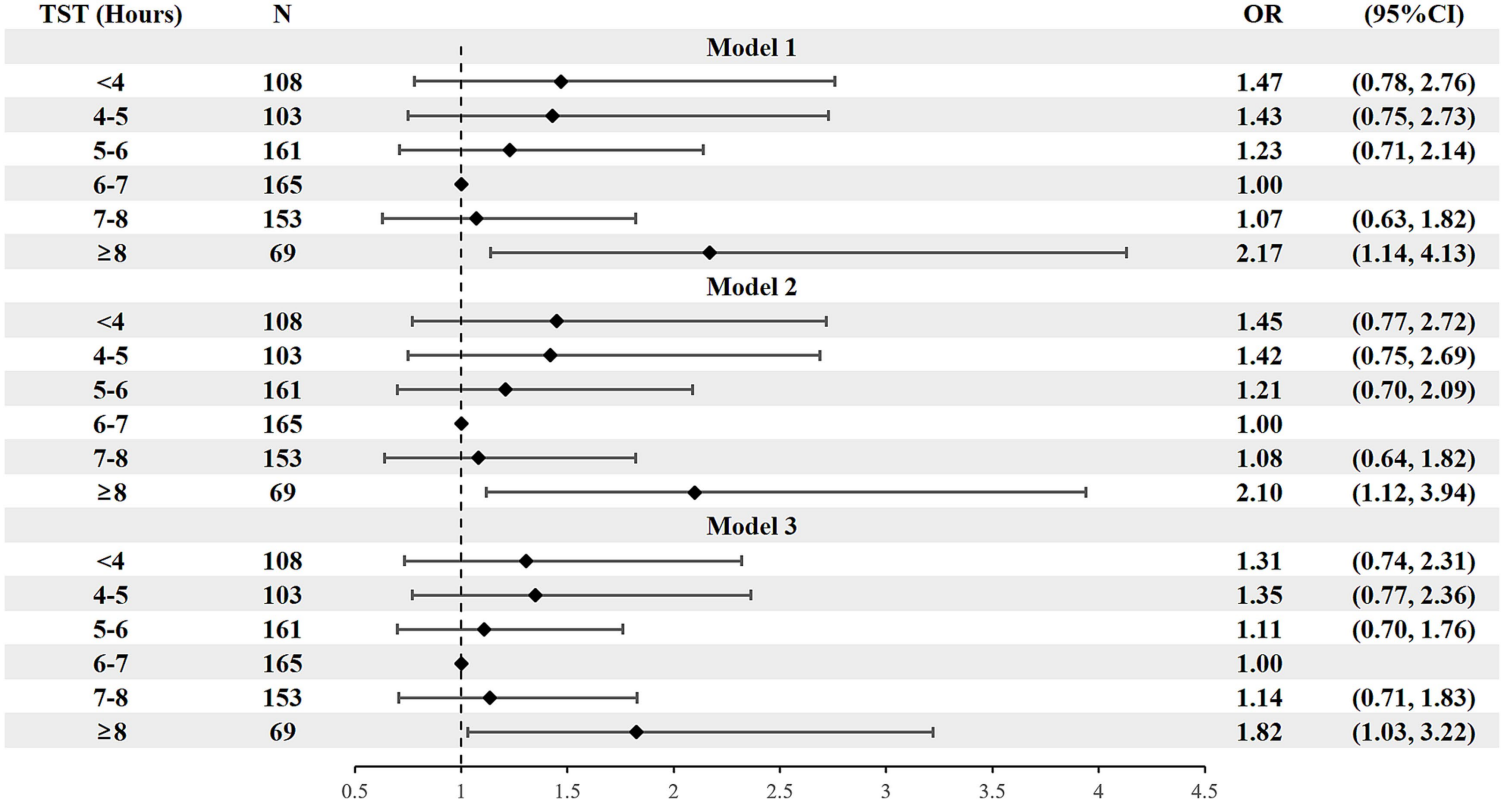
Forest plot of stepwise logistic regression analysis of TST with hypertension prevalence. TST, total sleep time. Model 1 adjusted for sex, age, residential area, and education level; Model 2 further adjusted for BMI, smoking status, alcohol consumption, and diabetes history; Model 3 additionally adjusted for AHI and sleep midpoint.

Model 1 demonstrated that both long and short SPT were significantly associated with a higher prevalence of hypertension. However, after further adjustment for confounding factors (Models 2 and 3), only short SPT (<6 h/night) remained a significant independent factor, while the association between long SPT and hypertension was no longer significant. In the fully adjusted model (Model 3), short SPT (<6 h/night) was associated with 2.05-fold higher odds of hypertension (OR = 2.05, 95% CI: 1.18–3.54; *p* = 0.011). For TST, only long TST group (≥8 h/night) was significantly associated with 1.82-fold higher odds of hypertension in model 3 (OR = 1.82, 95% CI: 1.03–3.22; *p* = 0.038) (Figures 1, 2).

### Nonlinear association of sleep duration and blood pressure levels

**Figure 3:**
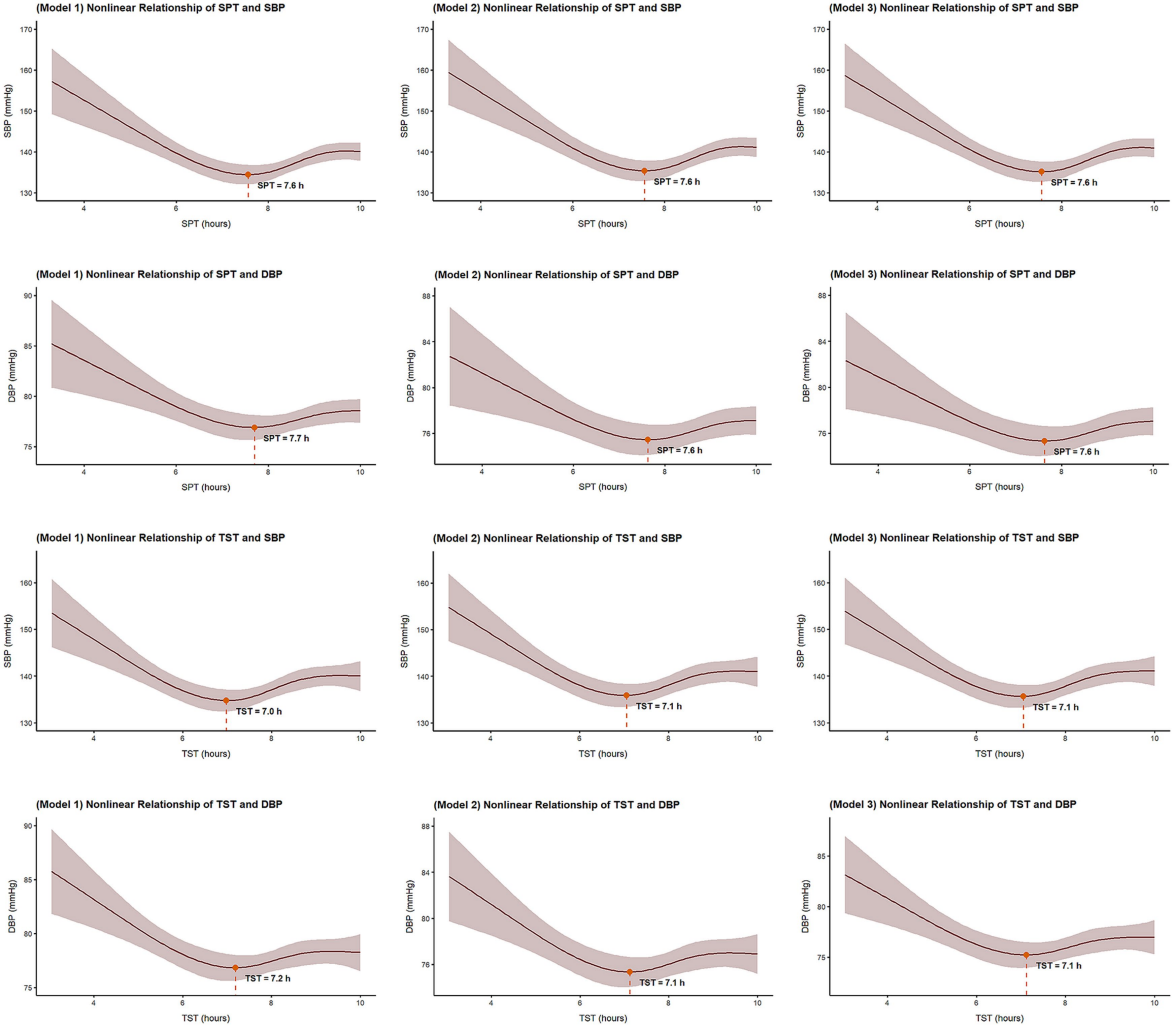
Restricted cubic spline plot of the nonlinear relationship of SPT, TST, and blood pressure levels. SPT, sleep period time; TST, total sleep time; SBP, systolic blood pressure; DBP, diastolic blood pressure. Model 1 adjusted for sex, age, residential area, and education level; Model 2 further adjusted for BMI, smoking status, alcohol consumption, and diabetes history; Model 3 additionally adjusted for AHI and sleep midpoint.

After adjusting for confounding factors, both SPT and TST exhibited significant nonlinear associations with SBP and DBP (*p* < 0.001). Asymmetric U-shaped associations between sleep duration and blood pressure levels were observed: Participants with shorter SPT and TST had markedly higher SBP and DBP, while longer sleep durations had modest elevations. For SPT, blood pressure nadirs occurred at 7.6–7.7 h for both SBP and DBP. Similarly, the lowest levels of blood pressure for TST were observed at 7.0–7.2 h across all adjusted models (Figure 3).

### Segmented linear regression of sleep duration and blood pressure levels

Using thresholds corresponding to the blood pressure nadirs (7.6 h for SPT and 7.1 h for TST), segmented linear regression models identified distinct linear associations between sleep duration and blood pressure. Below the threshold (SPT < 7.6 h), each 1-h increment in SPT was associated with a significant reduction in SBP (*β* = −2.94 mmHg, 95% CI: −4.82 – −1.06; *p* = 0.002) and DBP (*β* = −1.21 mmHg, 95% CI: −2.05 – −0.36; *p* = 0.005) in the fully adjusted Model 3. Conversely, in partially adjusted model 1, each 1-h increment in SPT above the threshold (SPT > 7.6 h) was associated with a 2.27 mmHg increase in SBP (95% CI: 0.15–4.39; *p* = 0.036). This association was no longer statistically significant in model 2 and model 3. Similarly, in partially adjusted Model 2, above the TST threshold (TST > 7.1 h), each additional hour of TST was associated with a 1.47 mmHg increase in DBP (95% CI 0.04 to 2.90, *p* = 0.044). Conversely, below this threshold (TST < 7.1 h), each additional hour was associated with a 0.74 mmHg decrease in DBP (95% CI −1.39 to −0.10, *p* = 0.025), although this association was no longer statistically significant in the fully adjusted Model 3 (Table 2).

**Table 2:** Segmented linear regression analysis of SPT, TST and blood pressure levels.

Sleep duration	Model	Below the Cutoff Point		Above the Cutoff Point	
		SBP (mmHg)	DBP (mmHg)	SBP (mmHg)	DBP (mmHg)
SPT (1 h increase)	Model 1	−3.48**	−1.35**	2.27*	0.56
		(−5.39, −1.56)	(−2.21, −0.50)	(0.15, 4.39)	(−0.77, 1.89)
	Model 2	−3.30**	−1.32**	1.84	0.32
		(−5.19, −1.41)	(−2.20, −0.43)	(−0.26, 3.93)	(−1.06, 1.71)
	Model 3	−2.94**	−1.21**	1.51	0.17
		(−4.82, −1.06)	(−2.05, −0.36)	(−0.61, 3.63)	(−1.19, 1.52)
TST (1 h increase)	Model 1	−0.99	−0.89**	1.62	1.55*
		(−2.22, 0.23)	(−1.55, −0.23)	(−0.91, 4.15)	(0.14, 2.96)
	Model 2	−0.78	−0.74*	1.56	1.47*
		(−2.23, 0.67)	(−1.39, −0.10)	(−0.96, 4.09)	(0.04, 2.90)
	Model3	−0.75	−0.52	1.39	1.36
		(−1.96, 0.45)	(−1.30, 0.25)	(−1.15, 3.94)	(−0.10, 2.82)

SPT, sleep period time; TST, total sleep time; SBP, systolic blood pressure; DBP, diastolic blood pressure.

Cutoff Point: SPT = 7.6 h, TST = 7.1 h.

*p < 0.05; **p < 0.01.

In subgroup analyses stratified by hypertension status (hypertension vs. non-hypertension), below the threshold (SPT < 7.6 h), SPT was significantly associated with reductions in SBP and DBP, in both subgroups. In the hypertension subgroup, the fully adjusted Model 3 showed that each 1-h increment in SPT was associated with a decrease in SBP (*β* = −3.16 mmHg, 95% CI: −4.85 to −1.47; *p* = 0.001) and DBP (*β* = −1.26 mmHg, 95% CI: −2.31 to −0.21; *p* = 0.019). A similar but weaker reduction was observed in the non-hypertension subgroup for SBP (*β* = −2.38 mmHg, 95% CI: −4.28 to −0.48; *p* = 0.014) and DBP (*β* = −1.08 mmHg, 95% CI: −2.09 to −0.07; *p* = 0.036). Additionally, different from the overall and non-hypertension groups, in the hypertension subgroup, below the threshold (TST < 7.1 h), each 1-h increment in TST was associated with a 0.85 mmHg reduction in DBP (95% CI: −1.79 to −0.09; *p* = 0.050) in Model 3 (Table 3).

**Table 3:** Segmented linear regression analysis of SPT and TST with blood pressure levels by hypertension status.

Hypertension status	*N*	Sleep duration	Below the Cutoff Point		Above the Cutoff Point	
			SBP (mmHg)	DBP (mmHg)	SBP (mmHg)	DBP (mmHg)
Hypertension	308	SPT	−3.16** (−4.85, −1.47)	−1.26* (−2.31, −0.21)	1.79 (−0.73, 4.31)	0.53 (−1.35, 2.41)
		TST	−1.14 (−2.69, 0.41)	−0.85* (−1.79, −0.09)	1.54 (−0.92, 4.00)	1.43 (−0.13, 2.99)
Non-hypertension	451	SPT	−2.38* (−4.28–0.48)	−1.08* (−2.09, −0.07)	1.42 (−1.68, 4.52)	0.36 (−1.58, 2.30)
		TST	−0.66 (−2.37, 1.05)	−0.43 (−1.65, 0.79)	1.23 (−1.81, 4.27)	1.29 (−0.41, 2.99)

Adjusted for sex, age, residential area (urban/rural), education level, BMI, smoking status, alcohol consumption, diabetes history, AHI, and sleep midpoint.

Cutoff Point: SPT = 7.6 h, TST = 7.1 h.

SPT, sleep period time; TST, total sleep time; SBP, systolic blood pressure; DBP, diastolic blood pressure.

*p < 0.05; **p < 0.01.

## Discussion

This study demonstrates an asymmetric U-shape association between HRV-Derived sleep duration and hypertension that differs from previous findings. In contrast to previous studies using self-reported sleep duration, which link long sleep to higher prevalence of hypertension (22, 33), we did not observe a higher prevalence of hypertension in individuals with SPT ≥ 9 h/night when using HRV-derived objective sleep measures. This discrepancy likely comes from bias in subjective sleep reporting, where individuals with hypertension may not separate excessive time in bed from actual sleep. Our objective monitoring approach effectively eliminated this measurement bias (34). However, we observed that longer TST was associated with a higher prevalence of hypertension compared with the reference group. This observation may indicate that sleeping longer than needed is more strongly associated with prevalence of hypertension, particularly in individuals who already obtain adequate sleep. This finding is consistent with the National Sleep Foundation’s sleep duration recommendations (34).

Consistent with previous studies using self-reported sleep duration, short SPT (<6 h) was associated with a two-fold higher prevalence of hypertension. However, the TST < 5 h group showed no significant association with hypertension prevalence, which is counterintuitive. One explanation is that SPT may serve as a more stable proxy for an individual’s habitual sleep patterns. In contrast, while TST objectively captures actual sleep on a given night, it exhibits greater night-to-night variability due to transient factors. Notably, initial acclimatization to the wearable device, even comfortably designed, may reduce TST on the first night, thereby compromising its representativeness of habitual sleep patterns. Furthermore, more extreme values in the TST < 5 h group likely contributed to greater data variability, thereby resulting in wider confidence intervals and a non-significant association.

This study demonstrates an asymmetric U-shaped association between sleep duration and blood pressure levels, and insufficient sleep exerts a more significant effect on blood pressure levels than prolonged sleep. However, this finding contrasts with the null linear relationships reported by Ramos et al. and Abbott et al. using actigraphy (35, 36). This discrepancy likely stems primarily from fundamental differences in analytical approaches to modeling sleep duration. Our use of restricted cubic splines and segmented linear regression was specifically intended to characterize U-shaped relationships in which both short and long sleep durations are associated with elevated blood pressure levels. In contrast, previous studies used conventional linear models, which can obscure or average out associations at the distribution extremes, potentially leading to a null finding even if a U-shaped relationship exists.

Notably, Wang et al. also applied restricted cubic splines in their study of patients with obstructive sleep apnea (OSA) (37). Their results demonstrated a linear negative correlation between PSG-derived sleep duration and hypertension prevalence, with no increased risk observed for longer sleep duration. This discrepancy may stem from differences in the study populations. Because of features such as intermittent nocturnal hypoxia and sleep fragmentation, patients with OSA typically experience poorer sleep quality and often have sleep deprivation even with long sleep duration, which may explain the absence of elevated risk with longer sleep duration (38). In contrast, our study included a sample of the general population, thus providing clearer insights into the correlations between prolonged sleep duration and both hypertension prevalence and blood pressure levels.

In studies using self-reported sleep duration, growing evidence indicates that both short and long sleep durations are associated with higher cardiometabolic risk, which aligns with the U-shaped relationship between objective sleep duration and blood pressure levels observed in our study (39, 40). The asymmetric U-shaped relationship is driven by distinct underlying mechanisms at each extreme. While short sleep may promote hypertension through direct physiological stress related pathways such as sympathetic overactivity and inflammation (41, 42), prevalent hypertension can also induce symptoms that lead to sleep fragmentation and reduced sleep duration. Conversely, long sleep is often an indicator of underlying conditions such as sleep apnea or cardiovascular disease which may contribute to both hypertension and elevated sleep requirements (43). Additionally, the fatigue and metabolic dysregulation related to hypertension itself may lead to prolonged sleep duration.

In this study, the optimal sleep durations for blood pressure were found to be 7.6 h for SPT and 7.1 h for TST. Recent research has similarly identified specific sleep durations associated with the most favorable blood pressure levels and the lowest risk of hypertension (40, 44). However, these findings are derived from self-reported sleep duration, and the inherent imprecision of such data in questionnaire means they tend to reflect approximate ranges (e.g., 7–8 h) rather than precise values. Our study overcomes this key limitation of previous studies by employing objective sleep monitoring, providing a clearly defined optimal sleep duration for blood pressure control. Additionally, Zhao et al. reported an asymmetric U-shaped relationship between sleep timing and hypertension prevalence, with optimal bedtime between 22:00–23:00 and wake-up time between 05:00–07:00 (45). Their focus on sleep rhythm complements our study’s focus on sleep duration, collectively suggesting that an optimal sleep pattern encompasses both dimensions. Further research is needed to establish the optimal sleep pattern among diverse populations.

By identifying an asymmetric U-shaped relationship and an optimal sleep duration, our findings move beyond merely establishing an association. This study provides a basis for designing future cohort studies and supports the application of portable sleep monitors in clinical implication. In primary care settings, simple single-lead ECG devices or other consumer-grade sleep monitors offer a readily accessible, actionable and continuously trackable metric for establishing an individual’s baseline sleep profile thereby facilitating the integration of objective sleep data into both cardiovascular risk assessment and hypertension management strategies.

Although the blood pressure changes per hour of sleep duration were modest in magnitude (0.74–2.94 mmHg), they may have a substantial public health impact at the population level. According to large-scale meta-analyses, each 1 mmHg reduction in SBP or 0.5 mmHg reduction in DBP is associated with 4% lower risk of stroke and a 2% lower risk of coronary heart disease (46). Furthermore, abnormal sleep duration is associated with other sleep parameters, such as sleep fragmentation, sleep efficiency, and AHI (47, 48). The combined effect of multiple sleep parameters may exert a clinically meaningful impact on blood pressure, exceeding that expected from sleep duration alone.

This study pioneers the use of HRV-based objective sleep metrics in hypertension research, mitigating recall bias associated with self-reported sleep data. We employed restricted cubic splines to characterize the non-linear relationship between sleep duration and blood pressure, and further identify key inflection points. Moreover, to quantify the magnitude of these effects, we used segmented linear regression to measure blood pressure changes associated with deviations from the optimal sleep duration. By precisely characterizing and quantifying this asymmetric U-shaped association between sleep duration and blood pressure, our findings offer direct clinical implications for blood pressure management and sleep guidance.

Five main limitations should be acknowledged. First, as a single-center study with participants having a mean age of 65.8 years, our findings may not be fully generalizable to younger populations with distinct sleep patterns. Second, it should be noted that SPT and TST segmented at inflection points showed non-normal distributions. While this reflects an inherent feature of segmented sleep duration parameters, this could potentially impact the validity of linear regression models. Third, the median 2-night monitoring period limited our ability to track sleep patterns over multiple nights—a key strength of wearable sleep monitoring. Consequently, we were unable to evaluate weekly variations in sleep duration, a known contributor to hypertension risk (49). Fourth, even with comfortably designed wearables, potential measurement effects of the wearable device and compromised model accuracy in individuals of severe sleep fragmentation may introduce measurement bias. Finally, as an observational study, our findings are unable to establish causal inferences.

## Conclusion

Wearable-derived objective sleep duration measurements demonstrate an asymmetric U-shaped association with both blood pressure levels and hypertension prevalence, with insufficient SPT and prolonged TST emerging as independent correlates of higher hypertension prevalence. Blood pressure nadirs were observed at 7.6–7.7 h for SPT and 7.0–7.2 h for TST, respectively.

## Data Availability

The datasets presented in this study can be found in online repositories. The names of the repository/repositories and accession number(s) can be found in the article/Supplementary material.
