# Supplementary material for: Asymmetric U-shaped association between hypertension and wearable-derived sleep duration
Source: Front Public Health. 2026 Jan 12;13:1724251. doi: 10.3389/fpubh.2025.1724251 (PMC12833621; doi:10.3389/fpubh.2025.1724251)
Supplement: Supplementary file 1 [file Table_1.docx]

## Supplement table 1: Comparative Analysis of Clinical and Subjective Sleep Parameters Between included and excluded participants

| Variables | Total | Included | Excluded | P value |
| --- | --- | --- | --- | --- |
| Participates, n | 1560 | 759 | 801 |  |
| Age, years | 66.09±10.47 | 65.83±10.40 | 66.34±10.52 | 0.371 |
| Gender |  |  |  | 0.518 |
| Men, n(%) | 579 (37.1) | 276 (36.4) | 303 (37.8) |  |
| Women, n(%) | 981 (62.9) | 483 (63.6) | 498 (62.2) |  |
| BMI, kg/m² | 23.99±2.87 | 23.96±2.86 | 24.02±2.88 | 0.683 |
| WC, cm | 83.71±9.13 | 83.63 ± 9.08 | 83.78±9.17 | 0.762 |
| WHpR | 0.90±0.08 | 0.90 ± 0.08 | 0.91±0.08 | 0.812 |
| WHtR | 0.54±0.06 | 0.54±0.06 | 0.54±0.06 | 0.739 |
| Blood Pressure |  |  |  |  |
| Systolic | 138.81±19.12 | 138.42±18.99 | 139.17±19.23 | 0.435 |
| Diastilic | 78.46±10.28 | 78.13±10.19 | 78.77±10.35 | 0.204 |
| Subjective Sleep Duration, hours | 6.58±1.09 | 7.31±1.32 | 6.55±1.11 | 0.281 |
| Subjective Sleep Midpoint, hours | 2.79±0.84 | 2.78±0.83 | 2.80±0.85 | 0.645 |

Data are presented as mean ± standard deviation or median (interquartile range) for continuous variables, and number (percentage) for categorical variables. BMI = body mass index; WC = waist circumference; WHpR = waist-to-hip ratio; WHtR = waist-to-height ratio; P-values derived from independent t-tests, Mann-Whitney U tests, or χ² tests as appropriate. Bold values indicate statistical significance (P < 0.05).
