# Supplementary figures and images for: Asymmetric U-shaped association between hypertension and wearable-derived sleep duration
Source: Front Public Health. 2026 Jan 12;13:1724251. doi: 10.3389/fpubh.2025.1724251 (PMC12833621; doi:10.3389/fpubh.2025.1724251)

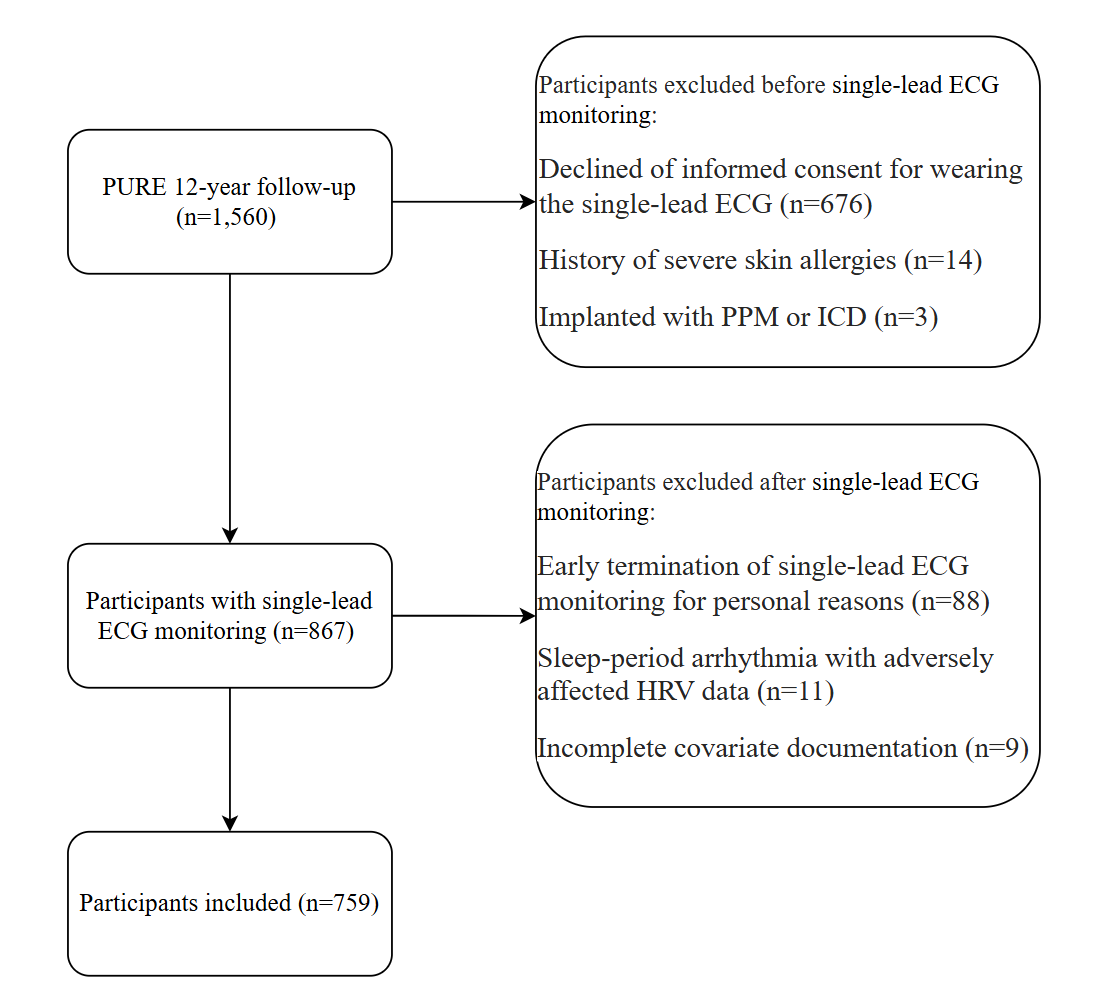

Supplement: Supplementary file 2 [file Image_1.png]
